# Extreme Interval Entropy Based on Symbolic Analysis and a Self-Adaptive Method

**DOI:** 10.3390/e21030238

**Published:** 2019-03-02

**Authors:** Zhuofei Xu, Yuxia Shi, Qinghai Zhao, Wei Li, Kai Liu

**Affiliations:** 1 Faculty of Printing Packaging Engineering and Digital Media Technology, Xi’an University of Technology, Xi’an 710048, China; 2 School of Mechanical and Precision Instrument Engineering, Xi’an University of Technology, Xi’an 710048, China

**Keywords:** empirical mode decomposition, empirical wavelet transform, intrinsic mode functions, symbolization, extreme interval entropy

## Abstract

Self-adaptive methods are recognized as important tools in signal process and analysis. A signal can be decomposed into a serious of new components with these mentioned methods, thus the amount of information is also increased. In order to use these components effectively, a feature set is used to describe them. With the development of pattern recognition, the analysis of self-adaptive components is becoming more intelligent and depend on feature sets. Thus, a new feature is proposed to express the signal based on the hidden property between extreme values. In this investigation, the components are first simplified through a symbolization method. The entropy analysis is incorporated into the establishment of the characteristics to describe those self-adaptive decomposition components according to the relationship between extreme values. Subsequently, Extreme Interval Entropy is proposed and used to realize the pattern recognition, with two typical self-adaptive methods, based on both Empirical Mode Decomposition (EMD) and Empirical Wavelet Transform (EWT). Later, extreme interval entropy is applied in two fault diagnosis experiments. One experiment is the fault diagnosis for rolling bearings with both different faults and damage degrees, the other experiment is about rolling bearing in a printing press. The effectiveness of the proposed method is evaluated in both experiments with K-means cluster. The accuracy rate of the fault diagnosis in rolling bearing is in the range of 75% through 100% using EMD, 95% through 100% using EWT. In the printing press experiment, the proposed method can reach 100% using EWT to distinguish the normal bearing (but cannot distinguish normal samples at different speeds), with fault bearing in 4 r/s and in 8 r/s. The fault samples are identified only according to a single proposed feature with EMD and EWT. Therefore, the extreme interval entropy is proved to be a reliable and effective tool for fault diagnosis and other similar applications.

## 1. Introduction

Self-adaptive methods are recognized as important tools in signal analysis of biomedical, seismic, geological and financial fields. Empirical mode decomposition (EMD) is a classical one self-adaptive method EMD, a widely used signal analysis method, is based on the simple assumption that any data consist of different simple intrinsic mode functions (IMF); therefore, a signal can be decomposed into a series of IMFs without any prior knowledge. Each IMF inherits different local characteristic time scales of a signal. After empirical mode decomposition, Hilbert Huang Transform (HHT) is always used for instantaneous amplitude, phase and frequency measurements. With EMD and HHT, signal analysis method has been enriched widely [1,2,3].

Since EMD has a strong adaptability, EMD is nearly suitable for all kinds of non-linear and non-stationary signals. According to IMFs from original signal, various signal analysis works can be realized directly such as filtering, noise reduction, decoupling, feature extraction and pattern recognition, etc. Currently, researchers and engineers have widely used EMD and its modified versions to detect and diagnose faults in machinery [4,5,6,7]. It has also been used to identify structural parameter changes and detect incipient faults [8]. In the analysis of biomedical and geological cases, EMD and similar methods are also can be used effectively [9,10,11,12].

With the improvement of EMD, related derivative methods have been proposed. To rectify and overcome the mode mixing effect in EMD, ensemble empirical mode decomposition (EEMD) has been presented [13,14]. Considering that the frequency of the added noise is hard to select in EEMD, an adaptive optimised EEMD (OEEMD) method, aiming to eliminate mode mixing better than EEMD, is presented and used in fault diagnosis more effectively [15]. Then, local mean decomposition (LMD), a new adaptive non-stationary signal processing method, is proposed. LMD decomposes multicomponent signals into single AM-FM signals which are termed product function (PF) components [16]. In addition, variational mode decomposition (VMD), developed to eliminate the mode mixing problem in EMD, has also gained some popularity in recent years as a promising technique for fault detection in various mechanical equipment [17,18].

Among these time-frequency methods, wavelet transform (WT), which can decompose a signal into several low high-frequency components and high-frequency components, has a good ability to show features of an abnormal signal. WT has more choices for a basis function to match a specific fault symptom, which is beneficial to fault feature extraction. Due to the advantages, WT has shown its tremendous usefulness in fault diagnosis of rotating machinery. In general, WT can be categorized as continuous wavelet transform, discrete wavelet transform and wavelet packet transform [7,19]. Consider that WT has a stronger ability of local frequency domain analysis for signals, another improved method, denoted as the empirical wavelet transform (EWT), was developed by Gilles [20]. In EWT, the frequency information of signal is extracted by the fast Fourier transform and a proper wavelet filter bank is established according to segmentations of the Fourier spectrum to decompose the pure vibration modes without mixture [21]. In many cases, the components obtained from EWT always have more advantages in signal analysis.

All methods mentioned above are always denoted as self-adaptive decomposition methods. A signal will be decomposed into a serious of new components with these mentioned methods; thus, the amount of information is also increased. In order to use these components effectively, a feature set is needed to describe them. With the development of artificial intelligence and pattern recognition, signal analysis is becoming more intelligent and dependent on feature sets [22,23]. Therefore, it is important to construct feature sets with low computational complexity with a strong representation ability in self-adaptive decomposition methods [5,6,24].

A new feature, extreme interval entropy, is proposed to realize the characterization of components obtained from self-adaptive decomposition in the research. This work is based on the symbolic analysis of extrema values. Extreme interval entropy given here can reveal the regularity of the relationship between positions of extreme values, thus it can describe the differences property in signals. The extreme interval entropy is applied to mechanical fault diagnosis successfully. Considering that EMD is a typical self-adaptive method and EWT has a better developing trend recently, they both were taken as main research objects in this work [25].

This paper is organized into four sections including the present one. In Section 1, an overview of the research purpose and content is given. Section 2 reviews the theory and principle involved in the proposed method. Symbolization methods based on extremum analysis and extremum interval entropy are also proposed. The technique is further validated using three series of experimental data from bearing faults in Section 3. Subsequently, this research is concluded in Section 4.

## 2. Symbolic Analysis for Components

### 2.1. Intrinsic Mode Functions

EMD is a powerful time-frequency domain analysis technique for decomposing a nonlinear and nonstationary time series into a set of orthogonal components named s IMFs. If the signal to be analyzed is x(t), the EMD process can be described as follows [1]:

(1) Find the positions and amplitudes of all local maxima and minima, then denote them as and correspondingly.
(2) Create the upper and lower envelopes by cubic spline interpolation of the local maxima and the local minima, respectively. Calculate the mean of both the upper and lower envelops;
(3) The envelope is then subtracted from the signal using. If satisfies the two conditions of IMF conditions as follows, it can be obtained as an IMF. Otherwise, set and repeat processes (1)–(3) until the residual satisfies the stopping criterion.
(4) Once IMF has been gotis obtained, should be replaced by the residual. The above process is repeated and the signal would be separated into *n* IMFs and a residue signal as in Equation (1). at last: Finally, the signal is decomposed into n number of IMFs and the residual signal.
  (1) $$x(t)=\sum_{i=1}^{n}c_{i}(t)+r_{n}(t)$$

An IMF is a function that satisfies the two following conditions: (i) in the whole data set, the number of extremes and the number of zero-crossings must either be equal or differ at most by one; (ii) at any point, the mean value of the envelope defined by local maxima and the envelope defined by the local minima is zero.

After converting a signal into the sum of a group components, the high-frequency and low-frequency components can be distinguished well. High frequency information is always contained in the former IMFs, while noise and low frequency information are in the residue signal and back IMFs. Therefore, the calculation and analysis are mainly focused on the first few components.

### 2.2. Symbolic Analysis for IMF

The symbolic analysis method is derived from nonlinear systems and it can simplify the signal to a large degree. In the symbolic method, the main trend of the signal is highlighted while local details are always ignored [26,27,28]. According to the definitions of EMD, the basic form of components is decided by extremum envelopes, and this means that the calculation is dependent on the positions of maximum and minimum values to a great extent.

**Figure 1:**
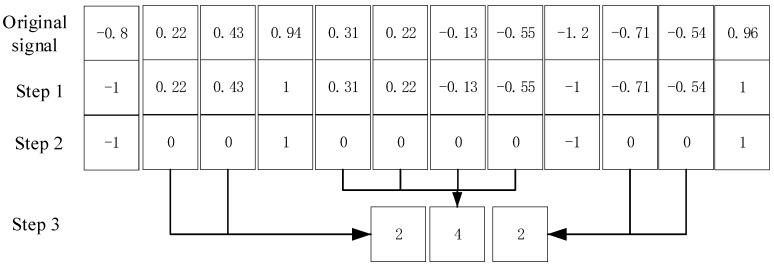
Symbolic analysis for the extreme value of IMF.

In order to obtain a high efficiency feature of IMF and eliminate redundant information, a new symbolic analysis method is proposed based on extrema values. The steps involved in the symbolic analysis are as follows,

(1) After the IMF component is obtained, set maximum values as 1 and minimum values as −1, and that means that the extremums are normalized.

(2) Set the values of non-extreme points as zero, and then there are only −1, 0 and 1 in the signals. Thus, IMF is changed into an integer sequence.

(3) Record the number of zero points between all adjacent extreme points one by one. Then a new sequence composed of these numbers can be obtained. This sequence can describe the relationship of the positions between adjacent extreme values in components effectively and is named the extreme interval sequence. One typical example is shown in Figure 1.

To avoid the application of complex features, the research attempts to use features as little as possible to realize the characterization of components. Among various signal features, entropy and similar features are often chosen, relying on their advantages in describing the signal as a whole system [29,30]. Thus, entropy is incorporated into the symbolic analysis and is named as Extreme Interval Entropy in this work. It is presented as Equation (2)

(2) $$E=-\sum_{i=1}^{k}p_{i}\log p_{i}$$

where $p_{i}$ is the appearance probability of those numbers with a value *i* in the extreme interval sequence. *k* is the maximum value in the extreme interval sequence and it represents the maximum distance between adjacent extreme values. As for the high-order components, high frequency means a short time interval; therefore, *k* in a high component is always smaller. On the contrary, *k* in lower component is much larger and does not need to be used, since the lower components have nearly no significance in analysis.

### 2.3. Symbolic Analysis of EWT

Symbolic analysis and extreme interval entropy can also be included in EWT, which has rapidly developed in recent years. In EWT, the frequency information of the vibration signal is extracted by the fast Fourier transform, and then a proper wavelet filter bank is also established due to segmentations of the Fourier spectrum to decompose without mixture. EWT has better performance than EMD in restraining the endpoint effect and model mixture. The properties of self-adaptive and wavelet transform are integrated into EWT [20,21,31].

EWT extracts the components, similar to IMFs in EMD, with a wavelet filter bank. These matched band pass filters are built around the peaks in the frequency spectrum. Take [0,π] as the range of normalized signal spectrum in calculation. Assuming that the signal is composed of *N* single components, then divide the spectrum into *N* continuous segments and there are (N+1) boundary lines determined. After the band division, a wavelet filter can be constructed to extract the band information and obtain the independent mode components step by step. Each segment is recorded as $\Lambda_{n}=[\begin{array}{cc} \omega_{n-1} & \omega_{n} \end{array}]$, where $\omega_{n-1}=0$ and $\omega_{n-1}=\pi$. Then, $\cup_{n=1}^{N}\Lambda_{n}=[\begin{array}{cc} 0 & \pi \end{array}]$. The empirical wavelets are defined as band pass filters on each $\Lambda_{n}$. Meyer’s wavelet is employed as the construction method in this work. For ∀n>0,n∈Z, the empirical scale function ${\overset{\wedge }{\varphi }}_{n}\left(\omega \right)$ and empirical wavelet function ${\overset{\wedge }{\psi }}_{n}\left(\omega \right)$ are defined according to the angular frequency ω and a function β(x) as follows.

(3) $${\overset{\wedge }{\varphi }}_{n}\left(\omega \right)=\left\{\begin{array}{l} 1\begin{array}{ccc} & & if \end{array}\left|\omega \right|\leq \left(1-\gamma \right)\omega_{n}\\ \cos\left[\frac{\pi }{2}\beta \left(\frac{1}{2\gamma \omega_{n}}\left(\left|\omega \right|-\left(1-\gamma \right)\omega_{n}\right)\right)\right]\begin{array}{cccc} & & if & \left(1-\gamma \right)\omega_{n}\leq \left|\omega \right|\leq \left(1+\gamma \right)\omega_{n} \end{array}\\ 0\mathrm{otherwise}, \end{array}\right\}$$

(4) $${\overset{\wedge }{\psi }}_{n}\left(\omega \right)=\left\{\begin{array}{l} 1\begin{array}{ccc} & & if \end{array}\left(1+\gamma \right)\omega_{n}\leq \left|\omega \right|\leq \left(1-\gamma \right)\omega_{n+1}\\ \cos\left[\frac{\pi }{2}\beta \left(\frac{1}{2\gamma \omega_{n+1}}\left(\left|\omega \right|-\left(1-\gamma \right)\omega_{n+1}\right)\right)\right]\\ \begin{array}{cccc} & & if & \left(1-\gamma \right)\omega_{n+1}\leq \left|\omega \right|\leq \left(1+\gamma \right)\omega_{n+1} \end{array}\\ \sin\left[\frac{\pi }{2}\beta \left(\frac{1}{2\gamma \omega_{n}}\left(\left|\omega \right|-\left(1-\gamma \right)\omega_{n}\right)\right)\right]\\ \begin{array}{cccc} & & if & \left(1-\gamma \right)\omega_{n}\leq \left|\omega \right|\leq \left(1+\gamma \right)\omega_{n} \end{array}\\ 0\mathrm{otherwise}, \end{array}\right\}$$

where β(x) represents the related function in Equation (5)

(5) $$\beta (x)=x^{4}(35-84x+70x^{2}-20x^{3})$$

γ is a parameter. Parameter γ can ensure that there is no overlap between two consecutive transition areas, so the parameter γ must meet the following condition in Equation (6).

(6) $$\gamma \leq \min_{n}\left[\frac{\omega_{n+1}-\omega_{n}}{\omega_{n+1}+\omega_{n}}\right]$$

According to the method of constructing wavelet transform, the detail coefficients are obtained by using the empirical wavelet function and the inner product of the original signal. The approximation coefficients are obtained by using the scaling function and the inner product of the original signal; respectively, the expression is as follows

(7) $$\omega^{\varepsilon }{}_{f}\left(n,t\right)=\left[f,\psi_{n}\right]=\int f\left(\tau \right)\bar{\psi \left(\tau -t\right)}d\tau =\left(\overset{\wedge }{f}\left(\omega \right)\bar{\overset{\wedge }{\psi_{n}}\left(\omega \right)}\right)$$

(8) $$\omega^{\varepsilon }{}_{f}\left(0,t\right)=\left[f,\varphi_{1}\right]=\int f\left(\tau \right)\bar{\varphi_{1}\left(\tau -t\right)}d\tau =\left(\overset{\wedge }{f}\left(\omega \right)\bar{\overset{\wedge }{\varphi_{1}}\left(\omega \right)}\right)$$

where $\omega^{\varepsilon }{}_{f}\left(n,t\right)$ represents the detail coefficients, and $\omega^{\varepsilon }{}_{f}\left(0,t\right)$ is the approximation coefficients. The result of the reconstructed original signal f(t) are written as follows

(9) $$\begin{array}{l} f\left(t\right)=\omega^{\varepsilon }{}_{f}\left(0,t\right)*\varphi_{1}\left(t\right)+\sum_{n=1}^{N}\omega^{\varepsilon }{}_{f}\left(n,t\right)*\psi_{n}\left(t\right)\\ =\left(\overset{\wedge }{\omega^{\varepsilon }{}_{f}}\left(0,\omega \right)\overset{\wedge }{\varphi_{1}}\left(\omega \right)+\sum_{n=1}^{N}\overset{\wedge }{\omega^{\varepsilon }{}_{f}}\left(n,\omega \right)*\overset{\wedge }{\psi_{n}}\left(\omega \right)\right) \end{array}$$

where $\overset{\wedge }{\omega^{\varepsilon }{}_{f}}\left(0,\omega \right)$ and $\overset{\wedge }{\omega^{\varepsilon }{}_{f}}\left(n,\omega \right)$ are Fourier transformations of $\omega^{\varepsilon }{}_{f}\left(0,t\right)$ and $\omega^{\varepsilon }{}_{f}\left(n,t\right)$, respectively. Empirical modal functions $f_{k}$ are defined as follows

(10) $$f_{0}\left(t\right)=\omega^{\varepsilon }{}_{f}\left(0,t\right)\varphi_{1}\left(t\right)$$

(11) $$f_{k}\left(t\right)=\omega^{\varepsilon }{}_{f}\left(k,t\right)\psi_{k}\left(t\right)$$

## 3. Experiment

**Figure 2:**
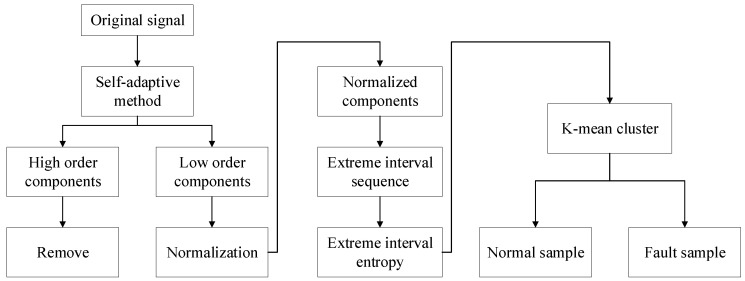
Flow chat of experiment.

In this part, extreme interval entropy is applied in two experiments of fault diagnosis with EMD and EWT, and K-mean cluster is selected to evaluate the performance of given features. According to Section 2, the experiment can be described as Figure 2. Firstly, the components from the self-adaptive method should be obtained such as IMF. Subsequently, the components are normalized, and the signal is restructured using −1, 0 and 1 only. Then, the extreme interval sequence is calculated for each normalized component, and K-mean cluster is introduced with extreme interval entropy for realizing the classification.

### 3.1. Fault Diagnosis of Rolling Bearing with a Frequency of 12 kHz

To verify the capability of the above proposed method, it is applied in the fault diagnosis of rolling bearings. The data of fault samples used for analysis came from the Case Western Reserve University Bearing Data Center [10,32]. Rolling bearings were mounted in a motor-driven rotating machinery system to support the motor shaft. Vibration data were collected using accelerometers, which were attached to the motor housing with magnetic bases, with a sampling frequency of 12 kHz and a rotating speed of 1797 r/min. The bearing type was a 6205-2RS-JEM SKF deep groove ball bearing. Motor bearings were seeded with faults using electro-discharge machining (EDM). Faults, 0.178 mm, 0.356 mm and 0.533 mm in diameter, were introduced at the inner raceway, rolling element and outer raceway separately. Samples with different faults were recorded in Table 1 named as A to J. Each sample in Table 1 has 10 groups of data.

**Figure 3:**
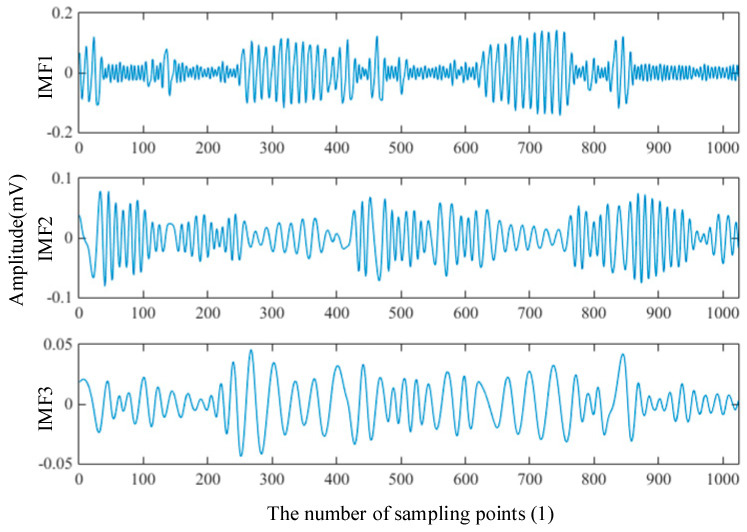
1st–3rd IMFs of normal bearing from EMD (12 kHz).

**Figure 4:**
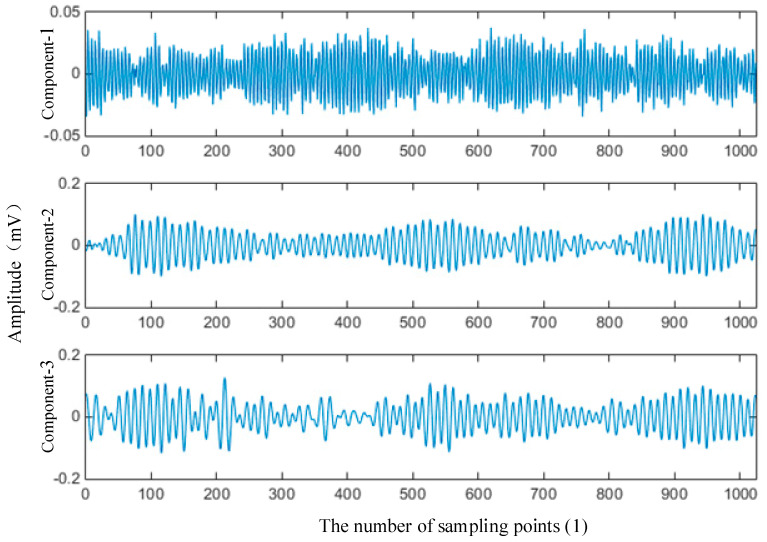
1st–3rd components of normal bearing from EWT (12 kHz).

The signal of normal bearing was selected as the standard signal. The first three components from EMD and EWT are shown in Figure 3 and Figure 4. It can be seen that although these two groups of components are different, they still have a similar trend. The energy and frequency are decreasing step by step.

**Figure 5:**
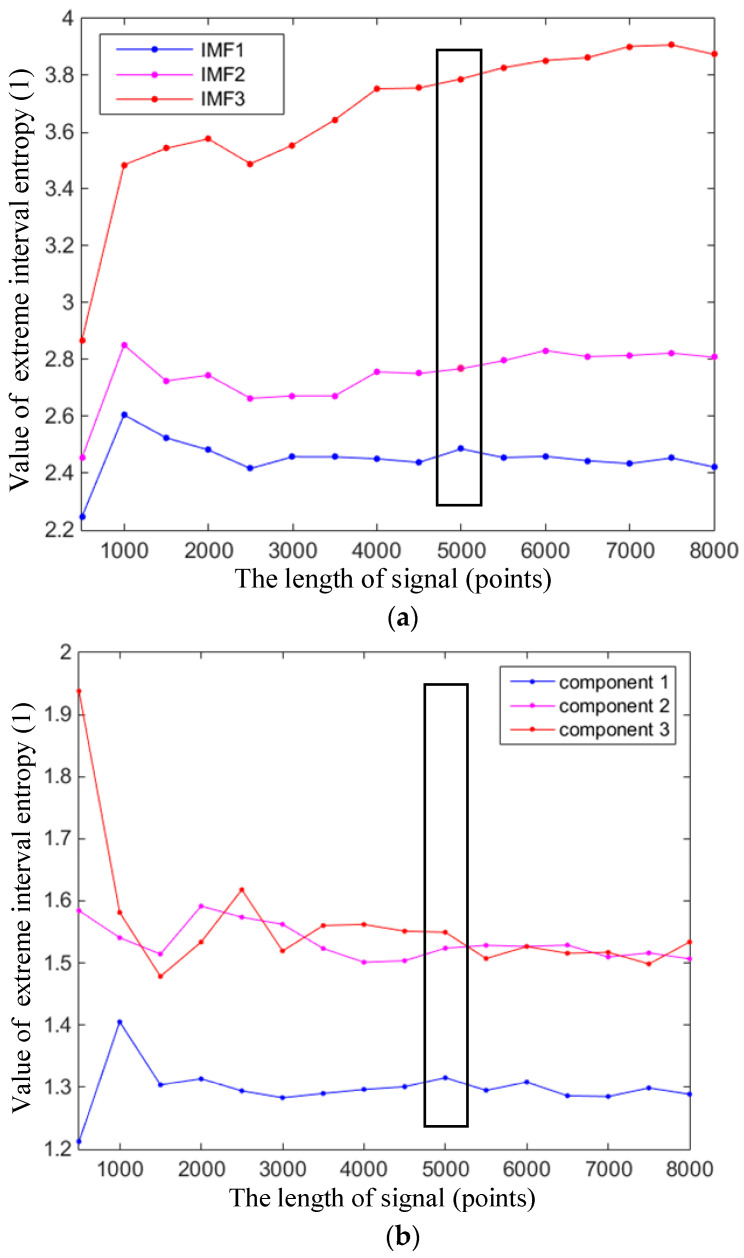
Extreme interval entropies with different lengths. (**a**) Extreme interval entropy changes with different lengths based on EMD (Sample-A, Component 1–3, EMD); (**b**) Extreme interval entropy changes with different lengths based on EWT (Sample-A, Component 1–3, EWT).

According to the definition of entropy, extreme interval entropy also changes with the length of a certain signal. If the signal is too short, the result will be insignificant because the information is not sufficient to calculate entropy-class statistical characteristics. However, when the signal is long enough, the value of extreme interval entropy will fluctuate in a small range and be treated as a fixed value approximately. In Figure 5, it can be seen how the values of sample-A change with different lengths. In order to obtain stable values in this experiment, a longer length is needed and 5000 points are used for each sample. Figure 5 shows that when the length exceeds about 4000, the extreme interval entropy will not fluctuate dramatically and will maintain a stable level. In order to be more stable, 5000 points were chosen and composed into a group of data in this section.

**Figure 6:**
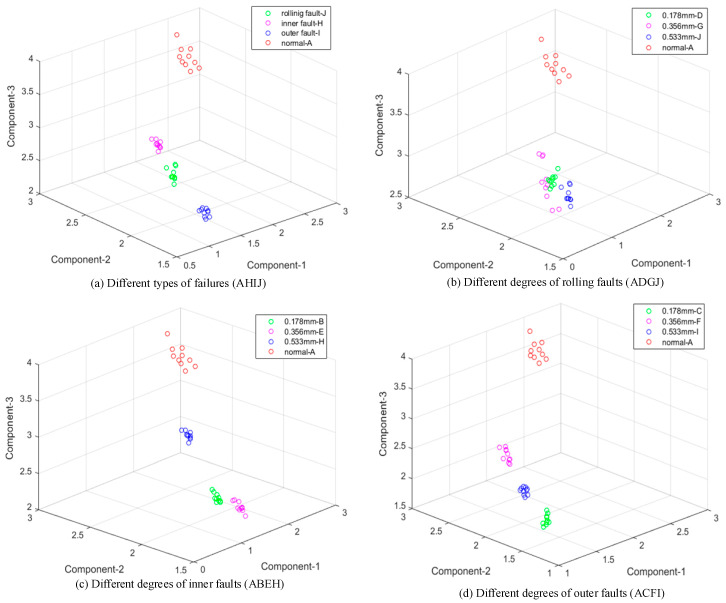
Result based on extreme interval entropy and EMD (12 kHz). (**a**) Different types of failures (AHIJ); (**b**) Different degrees of rolling faults (ADGJ); (**c**) Different degrees of inner faults (ABEH); (**d**) Different degrees of outer faults (ACFI).

**Figure 7:**
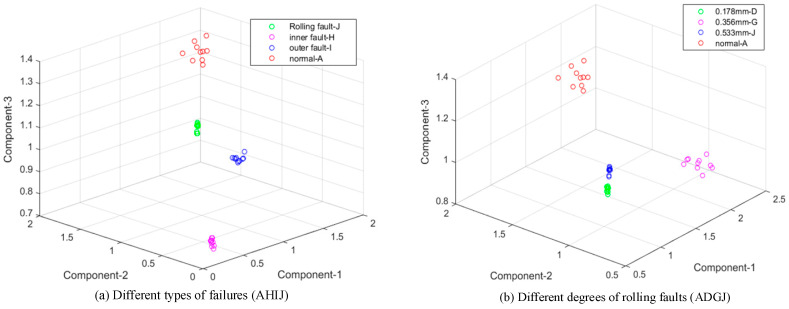

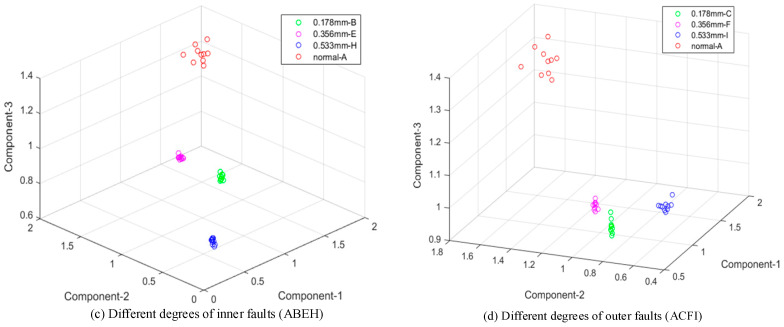
Result based on extreme interval entropy and EWT (12 kHz). (**a**) Different types of failures (AHIJ); (**b**) Different degrees of rolling faults (ADGJ); (**c**) Different degrees of inner faults (ABEH); (**d**) Different degrees of outer faults (ACFI).

The extreme interval entropies of all samples in Table 1 are calculated based on both EMD and EWT; then, their spatial distributions were drawn with the first three order extreme interval entropies as three-dimensional coordinates, recorded in Figure 6 and Figure 7. Compared with Figure 6a and Figure 7a, it can be seen clearly that the proposed feature has an ability to identify these four bearing faults obviously. Compared to Figure 6b–d and Figure 7b–d, it is found that the given method can identify faults with three different degrees and the normal sample well.

A normal bearing may have a higher entropy value in higher order components, and it means that normal bearing signals always have strong randomness but poor regularity, the same as many biological signals [10,33]. It can be found that extreme interval entropy has a similar property of general entropy-class features. Furthermore, the values decrease with the increase in the fault degree and this phenomenon is more obvious with EWT. In Figure 7b–d, a sample with a larger fault degree always concentrates in the lower value region more obviously than in Figure 6, and it is seen that extreme interval entropy with EWT is more effective in the identification of fault degrees than EMD.

According Figure 6 and Figure 7, it is clear that those samples can be distinguished directly due to the sensitivity of proposed features. The extreme interval entropies based on ETW and EMD are shown in Table 2. In order to prove the effectiveness, K-mean clustering is used to realize the classification. The K-mean clustering algorithm is one of the most popular data clustering approaches, which is applied in many practical applications such as statistical analysis, speech recognition, and genome data analysis. Since K-mean clustering, an unsupervised method, is a simple structure without strong nonlinearity as neural network, it is more suitable to reveal the classification capacity of features themselves. The accuracy rate is recorded in Table 3. Since the rate is nearly 100%, it can be concluded that extreme interval entropy has a strong ability in fault diagnosis of rolling bearing, and components from EWT are proved to be better in terms of recognition.

### 3.2. Fault Diagnosis of Rolling Bearing with a Frequency of 48 kHz

In an engineering environment, different sampling frequencies are used based on specific testing devices. In order to ensure the adaptability of the proposed method, the data with a sampling frequency of 48 kHz are chosen from the CWRU Bearing Data Center [32]. In general, the data with 48 kHz are harder to classify [34]. Test samples are shown in Table 4. Bearing faults were the same as in Section 3.1, thus still named A to J. Each sample has the same length and numbers as in Section 3.1.

**Figure 8:**
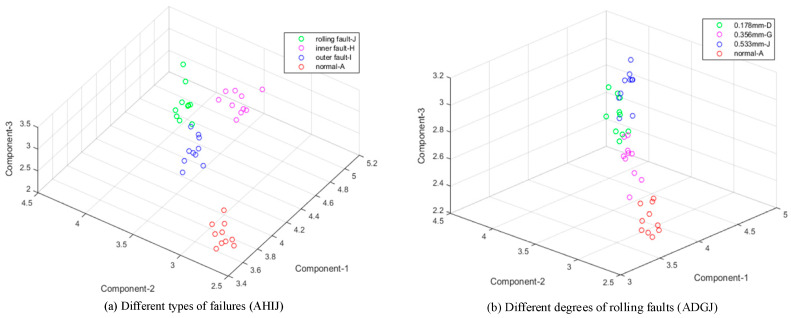

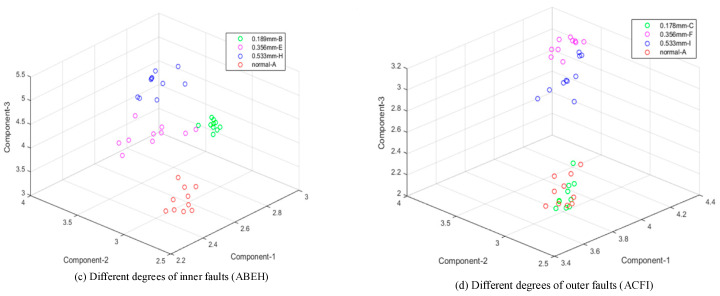
Result based on extreme interval entropy from EMD (48 kHz). (**a**) Different types of failures (AHIJ); (**b**) Different degrees of rolling faults (ADGJ); (**c**) Different degrees of inner faults (ABEH); (**d**) Different degrees of outer faults (ACFI).

**Figure 9:**
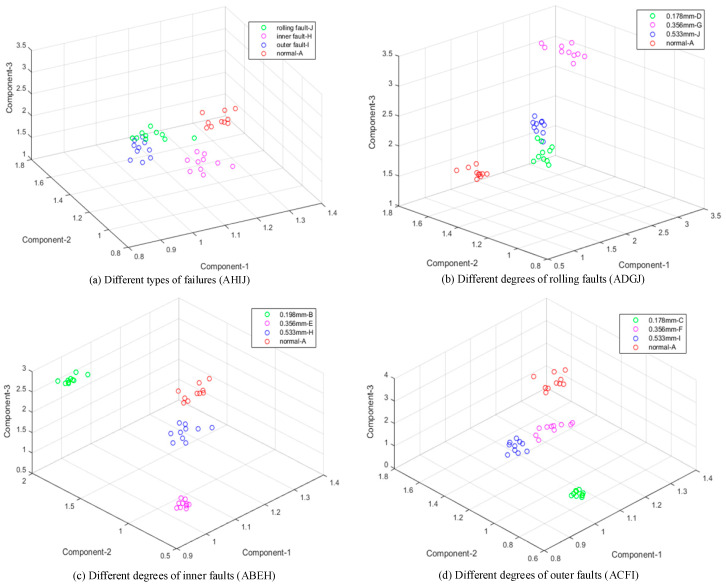
Result based on extreme interval entropy from EWT (48 kHz). (**a**) Different types of failures (AHIJ); (**b**) Different degrees of rolling faults (ADGJ); (**c**) Different degrees of inner faults (ABEH); (**d**) Different degrees of outer faults (ACFI).

The results are shown in Figure 8 and Figure 9 in the same form as in Figure 6 and Figure 7. In Figure 8, the result with 48 kHz is worse and more chaotic than samples with 12 kHz based on EMD. However, the distribution in Figure 9 still keeps four obvious areas with extreme interval entropies from EWT, and samples of various degrees and classes were all identified well. With K-means cluster, accuracy rates based on EMD and EWT reach 75%–100% and 95% to 100%, respectively, in Table 5. 

Compared to Section 3.1 and Table 4, the result is worse, as expected, while the accuracy rate still keeps a high level for given features. Four samples were identified to a 100% rate with K-means cluster. Therefore, these two experiments both proposed methods that can be used in fault diagnosis of rolling bearings under different frequencies effectively.

### 3.3. Fault Diagnosis of Rolling Bearing in Printing Press

**Figure 10:**
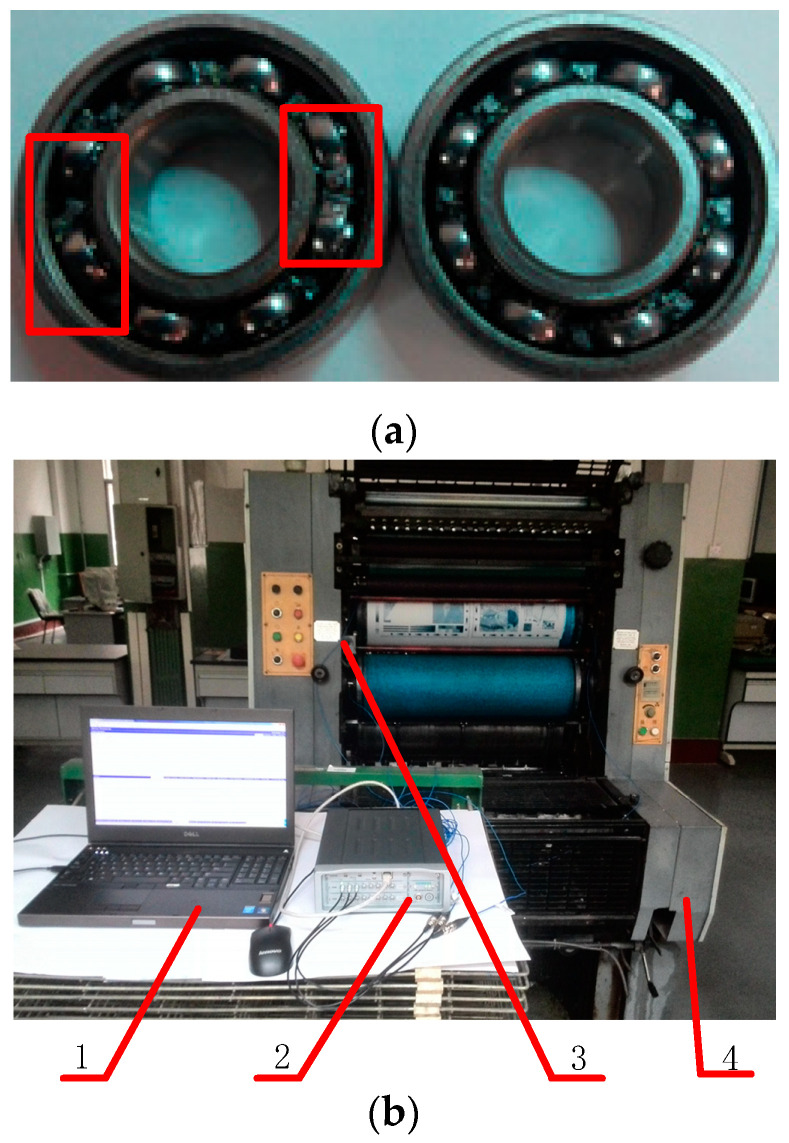
Experimental equipment and testing device. (**a**) Testing bearing; (**b**) Experimental equipment, (1) Workstation; (2) Data acquisition device; (3) Accelerometer; (4) Printing equipment.

The proposed method was applied to the fault diagnosis of rolling bearing in a printing press. A printing press for offset, GUANGHUA 650, was used. The testing bearing was NSK 6001Z mounted on the both ends of ink rollers and water rollers of the equipment. The bearing fault in a printing press is normally easy to be damaged since the printing process involves a large number of chemical solvents such as ink, alcohol, embossing liquid, etc. The printing pressure also shortens the service life during the contact between rollers. As a result, the main fault of rolling bearings in this condition was the roller misalignment caused by cage damage under chemical and pressure actions. This kind of fault is different from the experiment in Section 3.1 and Section 3.2 [35,36]. In this experiment, fault samples were made artificially to simulate the working condition, and the cage fault of rolling bearing was caused by an electro-discharge machining. The rolling bearings used in experiment are shown in Figure 10a; the bearing with a damaged cage is on the left while a normal one is on the other side, and the typical fault area has been marked with red color.

**Figure 11:**
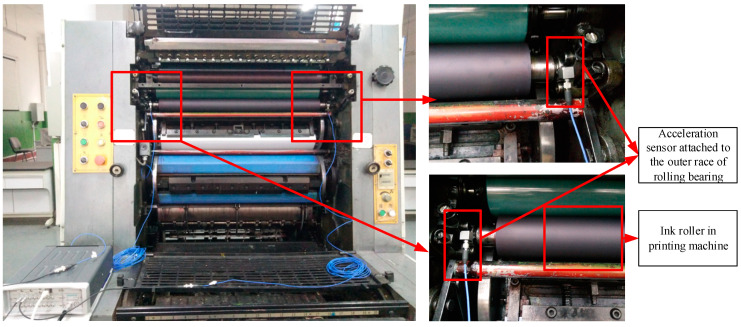
The position of the acceleration sensor and rolling bearing.

The experimental rig is shown in Figure 10b. The vibration signals were obtained with acceleration sensors. The acceleration used here is PCB 333B30 and serial number LW55675, with a sensitivity of 100 mV/g. In order to collect and calculate the data, LMS measurement system is used which contained a SCM202 chassis, the LMS-SCM-V8-E data acquisition card and an industrial computer Dell-M4800. In this experiment, the acceleration sensor was attached to the outer race of the bearing as shown in Figure 11. The bearing is used for the ink roller and is mounted on both ends. The accelerometer recorded the vibration in the normal direction of the outer ring. The sampling frequency is set to 50 kHz. The printing speed was set as 3600 per/hour and 7200 per/hour, and corresponding speeds of bearings were 4 rad/s and 8 rad/s, respectively, according to the rotation ratio.

**Figure 12:**
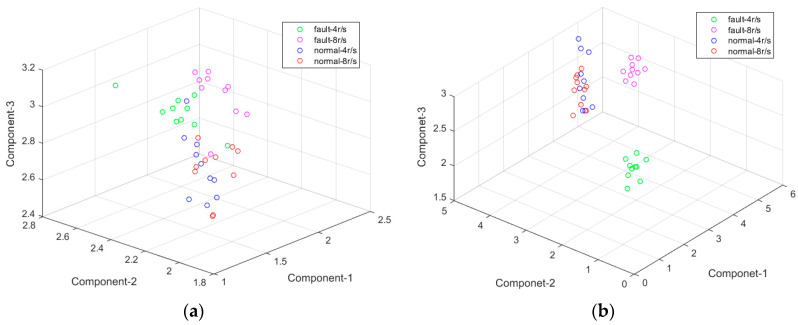
The extreme interval entropies based on (**a**) EMD and (**b**) EWT.

A printing press is a highly integrated piece of equipment, and its noise interference in testing is much stronger than during a regular bearing failure experiment. Extreme interval entropies based on both EMD and EWT were used under two speeds. The results are shown in Figure 12. From these two figures, the features with EMD cannot be identified directly, while EWT can identify not only the fault but also the fault under different speeds correctly. The normal states under different speed cannot be identified, since there is nearly no impact or vibration in them.

**Figure 13:**
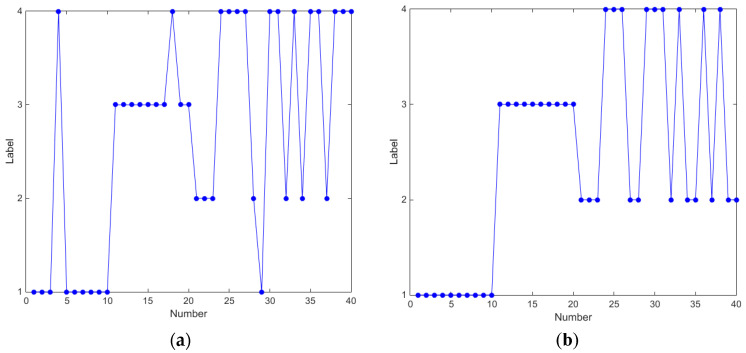
Result of clustering based on E (**a**) EMD and (**b**) EWT.

The result with K-means cluster is recorded in Table 6. In sample B, the accuracy rate is only 72.5% and 77.5%, and this is because it is hard to distinguish the normal samples under different speeds. In Sample A of Table 6, the normal samples under both 4 r/s and 8 r/s were treated as a whole sample; therefore, the accuracy rate can reach 95% and 100% with EMD and EWT. The cluster results are shown in Figure 13. Labels on the Y axis represent different samples and the Number axis represents the number of samples. No.1–No.10 are fault bearings in 4 r/s and No.11–No.20 is 8 r/s. No.21–No.30 are normal bearings in 4 r/s and No.31–No.40 is 8 r/s. EWT still shows a better result.

## 4. Conclusions

In this paper, extreme interval entropy was proposed to describe the components, from self-adaptive method, based on their hidden property. The given method was incorporated using EMD and EWT, and then applied in fault diagnosis for rolling bearing. Two different experiments were used, and various faults of rolling bearing were identified successfully using 1st–3rd high order components. The k-means cluster was used to evaluate the features. The main conclusions are as follows:

(1) A symbolized method was given to normalize the components from a self-adaptive perspective according to the positions of extreme values. With the symbolization, the components were simplified toin a large degree (only contains 1, −1, 0). Then, an improved feature for these simplified components, extreme interval entropy, was proposed and calculated for to similar self-adaptive components.
(2) According to the identified result of three group experiments, the extreme interval entropies of high order components can be distinguished in a 3D figure. Both different fault kinds and degrees were distinguished well in Section 3.1 and Section 3.2 by the given method under sample frequencies of 12 kHz and 48 kHz. Extreme interval entropy was proved to be an effectively feature in this fault recognition. A cage fault in the rolling bearing of a printing press was also identified correctly in Section 3.3. Extreme interval entropy with EWT always has a better effect.
(3) The effectiveness of the proposed method was evaluated with K-means cluster. The accuracy rate of fault diagnosis in rolling bearing was between 75% to 100% with EMD while 95% to 100% with EWT. In the experiment with thein printing press, the given method could reach to a 100% accuracy rate with EWT in identification of the normal bearing, fault bearing in 4 r/s and in 8 r/s (cannot distinguish normal samples atin different speeds). Extreme interval entropy was proved to be a reliable and effective tool for fault diagnosis and other similar applications.

In our future research, extreme interval entropy will be applied to more complex conditions such as complex mechanical failures. Since self-adaptive methods have developed quickly in recent years, extreme interval entropy will be promoted to obtain effective features for those new improved self-adaptive methods in signal processing. Moreover, the given method can be integrated into pattern recognition as an input vector to realize the identity of specific signals.

## Figures and Tables

**Table 1:** Test samples of rolling bearing with different faults (12 kHz).

Sample	Bearing Condition	Diameter of Faults (mm)
A	Normal	---
B	Inner fault	0.178
C	Outer fault	0.178
D	Rolling fault	0.178
E	Inner fault	0.356
F	Outer fault	0.356
G	Rolling fault	0.356
H	Inner fault	0.533
I	Outer fault	0.533
J	Rolling fault	0.533

**Table 2:** Extreme interval entropy based on EMD and EWT (12 kHz).

Order	Sample	EMD	EWT	Sample	EMD	EWT
1st	A	2.404	1.310	F	1.227	1.013
	B	1.258	1.015	G	0.986	0.965
	C	1.174	0.972	H	0.807	0.736
	D	0.921	1.058	I	1.231	1.001
	E	1.215	1.054	J	1.130	1.158
2nd	A	2.725	1.564	F	1.942	0.966
	B	1.849	0.444	G	2.129	0.783
	C	1.343	0.825	H	1.924	0.283
	D	2.039	0.817	I	1.702	0.590
	E	1.609	0.878	J	1.928	0.785
3rd	A	3.619	1.539	F	2.787	0.817
	B	2.388	0.669	G	2.867	1.999
	C	2.018	0.721	H	3.328	0.402
	D	2.945	0.754	I	2.302	1.043
	E	2.387	0.602	J	2.740	0.730

**Table 3:** Accuracy rate of K-means cluster based on EMD and EWT (12 kHz).

Sample	EMD	EWT
A, H, I, J	100.00%	100.00%
A, D, G, J	82.50%	100.00%
A, B, E, H	100.00%	100.00%
A, C, F, I	100.00%	100.00%

**Table 4:** Test samples of rolling bearing with different faults (48 kHz).

Sample	Bearing Condition	Diameter of Faults (mm)
A	Normal	---
B	Inner fault	0.178
C	Outer fault	0.178
D	Rolling fault	0.178
E	Inner fault	0.356
F	Outer fault	0.356
G	Rolling fault	0.356
H	Inner fault	0.533
I	Outer fault	0.533
J	Rolling fault	0.533

**Table 5:** Accuracy rate of K-means cluster based on EMD and EWT (48 kHz).

Sample	EMD	EWT
A, H, I, J	100.00%	95.00%
A, D, G, J	75.00%	97.50%
A, B, E, H	95.00%	100.00%
A, C, F, I	75.00%	100.00%

**Table 6:** Accuracy rate of the K-means cluster in the printing press.

Sample		EMD	EWT
A	Fault 4 r/s Fault 8 r/s Normal	95.00%	100.00%
B	Fault 4 r/s Fault 8 r/s Normal 4 r/s Normal 8 r/s	72.50%	77.50%
